# Effectiveness of ART and Condom Use for Prevention of Sexual HIV Transmission in Serodiscordant Couples: A Systematic Review and Meta-Analysis

**DOI:** 10.1371/journal.pone.0111175

**Published:** 2014-11-04

**Authors:** Huixin Liu, Yingying Su, Lin Zhu, Jiannan Xing, Jing Wu, Ning Wang

**Affiliations:** National Center for AIDS/STD Control and Prevention, Chinese Center for Disease Control and Prevention, Beijing, China; University of Ottawa, Canada

## Abstract

**Background:**

Consistent and correct condom use and suppressive antiretroviral therapy for the infected partner are two of the primary strategies recommended for prevention of heterosexual HIV transmission in serodiscordant couples today. The applied effectiveness of treatment as a prevention strategy in China is still under investigation, and much less is known about its effects in the presence of other prevention strategies such as consistent condom use.

**Methods:**

We conducted a systematic search in PubMed and three Chinese language databases to identify relevant articles for the estimation of relative effectiveness of a) consistent condom use and b) ART use by index partners for preventing HIV transmission in serodiscordant couples. We also estimated the prevention effectiveness of ART stratified by condom use level and the prevention effectiveness of consistent condom use stratified by ART use level.

**Results:**

Pooled results from the eleven eligible studies found a pooled HIV seroconversion incidence of 0.92 cases per 100 person years (PY) among HIV-negative spouses whose index partners were taking ART versus 2.45 cases per 100 PY in untreated couples. The IRR comparing seroconversion in couples where the index-partner was on ART versus not on ART was 0.47 (95%CI: 0.43, 0.52), while stratified by condom use, the IRR was 0.33(0.17,0.64). The IRR comparing incidence in couples reporting “consistent condom use” versus those reporting otherwise was 0.02(95%CI:0.01,0.04), after stratified by ART use level, the IRR was 0.01(95%CI: 0.00, 0.06).

**Conclusions:**

ART use by index partners could reduce HIV transmission in serodiscordant couples, and the effectiveness of this prevention strategy could be further increased with consistent condom use.

## Introduction

The probability of HIV transmission through sexual intercourse is broadly associated with factors including type of sexual intercourse [1], infection stage of the infected partner [2], susceptibility of the uninfected partner [2], [3] and viral load in genital secretions of the infected person [4], [5]. Estimates of the level of protection against HIV transmission from consistent and correct condom use has ranged from 60% to 96% [6], [7]. Antiretroviral treatment (ART) to suppress seminal and vaginal HIV-1 shedding [8], [9] has long been hypothesized to explain the lower rates of transmission in treated HIV serodiscordant couples (in which only one partner is HIV infected) [10], [11], populations with higher ART coverage[12]–[14], and through mathematic models [15]–[17]. A randomized clinical trial in 2011 [18] provided definitive proof that ART can nearly eliminate HIV transmission risk, but this reflects efficacy under optimal conditions of a clinical trial. Much research now focuses on the effectiveness (as opposed to efficacy) of ART as it is administered in real world settings, particularly in resource poor areas with limited access to early ART or strong adherence support [19]. In addition, simultaneous use of multiple behaviorally modified prevention methods such as condoms and ART is likely not uncommon and can be expected to modify effect of competing methods, both biologically and as mediated by user adherence. A better understanding of the effectiveness of these two prevention methods in a population of HIV serodiscordant couples can provide much needed insight into prevention programming and counseling in similar settings.

Since 2009, heterosexual transmission has overtaken injection drug use as the dominant mode of HIV transmission in China [20], many cases are thought to be from stable, HIV-serodiscordant relationships [21] making prevention of HIV transmission in this population a public health priority. In addition, condom use for HIV prevention and universal access to free ART for HIV infected individuals are two major priorities of China's HIV/AIDS control policy. The massive support from the central government for these programs therefore provides unique insight into the separate and combined effects of these methods on heterosexual HIV transmission. China's earliest HIV intervention programs launched in the early 2000's focused on condom promotion and sexual behavior change among key risk groups including female sex workers, followed by a period of massive scale up in the general population including HIV serodiscordant couples [6]. China's free national antiretroviral therapy program (NFATP) began in 2002 and within five years over 52 thousand patients were enrolled in the program, providing life-saving care and treatment to an estimated 97% of treated HIV patients in China. Despite wide regional disparity in availability of services such as routine laboratory monitoring, adherence counseling, or drug resistance testing, the NFATP has achieved nearly universal ART access and greatly reduced mortality from 39.3 to 14.2 deaths per 100 PY since its inception to 2009 [22].

This meta-analysis synthesized information from the published literature on China's HIV serodiscordant couples cohort to summarize HIV-1 seroconversion incidence rates of uninfected partner in serodiscordant couples by level of ART and condom use. We also estimated the protection effectiveness of ART and consistent condom use in reducing HIV-1 transmission in serodiscordant couples.

## Methods

### Search strategy and Study Selection

This study was conducted and reported according to the Preferred Reporting Items for Systematic Reviews and Meta-Analyses (PRISMA) statement which was issued in 2009 [23], we conducted a systematic search in both English and Chinese in PubMed and three Chinese databases including Wanfang Database, China National Knowledge Infrastructure (CNKI) and Chinese Biomedical Literature Database (CBM), from inception to March 31,2014. The following search string was used to search for relevant articles: ("HIV"[All Fields] OR "human immunodeficiency virus"[All Fields] OR "AIDS"[All Fields] OR "acquired immunodeficiency syndrome"[All Fields]) AND (Couples[title/abstract] OR (sex*[title/abstract] AND partner*[title/abstract]) OR spouse*[title/abstract] OR dyad*[title/abstract] OR married[title/abstract] OR marital[title/abstract] OR “Marriage” [MeSH] OR “spouses” [MeSH] OR serodiscord* [title/abstract] OR sero-discord* [title/abstract] OR discord*[title/abstract]) AND (transmission[title/abstract] OR infection [title/abstract] OR infectivity[title/abstract] OR infectiousness[title/abstract]) AND (China [title/abstract] OR Chinese[title/abstract]).(Search strings used for Chinese language searches are available from the authors on request).

Two independent reviewers (HXL and LZ) performed an initial screen of titles and abstracts for basic relevance, followed by a full-text examination to which the following inclusion and exclusion criteria were applied. Any discordant findings between the two reviewers were adjudicated by a third reviewer(NW). Studies that were included were 1) longitudinal; 2) included follow-up of stable heterosexual partners in which one partner was confirmed HIV positive(index-partner) and the other confirmed HIV negative (HIV-negative spouses); and 3) provided information on HIV seroconversion incidence among HIV-negative spouses. Articles were excluded if they did not have information on duration of HIV exposure of HIV-negative spouses; or did not provide information on ART status of the index-partner. Articles using the same study population were selected based on whichever had the larger sample size or provided greater detail on relevant measures.

### Data extraction and quality assessment

The following information was extracted from the eleven identified eligible studies: author names, year of publication, study period, sample size and follow-up rates, ART exposure in index-partners, sex of index partners, HIV seroconversion in HIV-negative spouses, reported frequency of sexual intercourse, reported condom use status. Where possible, observations of couples reporting no sexual intercourse were excluded from the analysis. The Newcastle-Ottawa Scale for cohort study (NOS) [24] was used to assessing the quality of the eligible studies, which contains eight items (1 point per item, provides a score ranging from 0-8 points), categorized into sample selection, comparability and outcome dimensions.

### Statistical analysis

The main outcomes were 1) pooled incidence rates of HIV seroconversion among the HIV-negative spouses by ART and condom use level, the time to seroconversion of the HIV-negative spouses was defined as the midpoint between the last negative and first positive result of HIV-antibody test as each study reported; 2) where data were available, we estimated the crude incidence risk ratio (IRR) of HIV seroconversion comparing ART use among the index-partner with no ART use, and the IRR of HIV seroconversion among different condom use level couples; 3) where data were available, we estimated the adjusted prevention effectiveness of ART stratified by condom use level, and the adjusted prevention effectiveness of consistent condom use stratified by ART use level; 4)we also stratified analyses by duration of follow up (less than two years versus two years or more), studies which stratified by index-partners' ART use status to find out whether the prevention effectiveness of ART were durable as time went on. Condom use was defined as “consistent” if couples responded to questions of their condom use behavior in the most recent time period(past 12 months) as “consistent condom use” Those responding otherwise “not consistent”, “not always”, ”seldom” or “never” were classified as “non-consistent” users.

Cochran's *Q* statistic and the *I^2^* statistic were used to assess the heterogeneity, and used a cutoff value of p<0.1 for *Q* statistic and values of 50% for *I^2^* statistic to determine statistical significance for determining heterogeneity. Random-effects Poisson regression models were used to estimate the pooled HIV-1 seroconversion incidence with 95% confidence intervals (95% CI). For each study, the total number of events was considered to be Poisson distributed for a given sum of person-year, and the total exposure time was used as an offset variable, with γ-distributed random effects on the study level [25]. All analyses were conducted using R [26], STATA version 12 (Stata Corporation, College Station, Texas, USA) and the Cochrane Collaboration' Review Manger 5.2 software package.

## Results

### Study population

**Figure 1:**
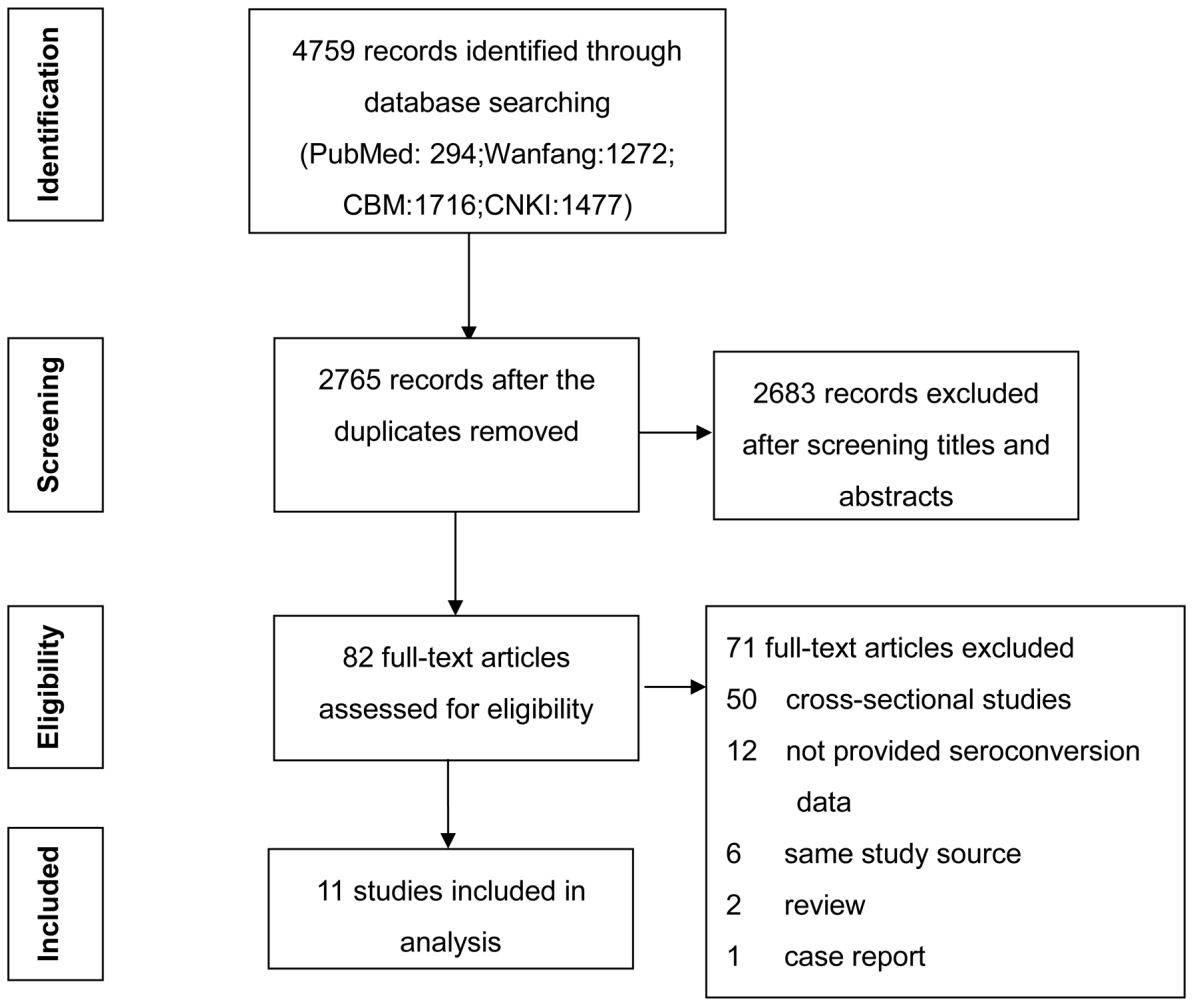
Flow chart of the process of studies selection.

Of the 4759 articles identified by our search, 2765 non-duplicate articles underwent a titles and abstracts screening. The remaining 82 articles were then submitted to full text review to determine eligibility according to the inclusion criteria, leaving 11 studies after the others were excluded for cross-sectional study design or lack of HIV negative partners' seroconversion outcomes [27] (Figure 1). The final 11 studies included information on 45615 HIV serodiscordant couples, most of which were followed in southern Yunnan and central Henan provinces. Characteristics of articles included in this analysis are summarized in Table 1.

**Table 1:** Study characteristics.

Author (year)	Province	Sample size (N of couples)	Study period	Total person-year	No. of seroconversions in non-index partner
Zheng Xiwen 1993 [31]	Yunnan	62	1990–1992	122	6
Zheng Xiwen 1994 [32]	Yunnan	48	1992–1993	48	1
Zhang Ke 2001 [33]	Beijing*	40	2000	20	0
Mao Yurong 2004 [34]	Xinjiang	22	1997–2000	24.625	8
Li Jingyun 2006 [35]	Henan	52	2002–2005	87.25	0
Qiao Xiaochun 2009 [36]	Shanxi	64	2003–2005	128	1
Pan Xiaohong 2010 [37]	Zhejiang	75	2007–2009	143.5	3
Duan Song 2010 [38]	Yunnan	790	2005–2008	1202.35	31
Jia Zhongwei 2012 [28]	National	38862	2003–2011	101295.1	1631
He Na 2013 [21]	Yunnan	1101	2009–2011	1127	17
Wang Lan 2013 [39]	Henan	4499	2008–2011	12134.55	100

*A provincial level city.

### Pooled incidence rates of HIV-1 seroconversion

**Figure 2:**
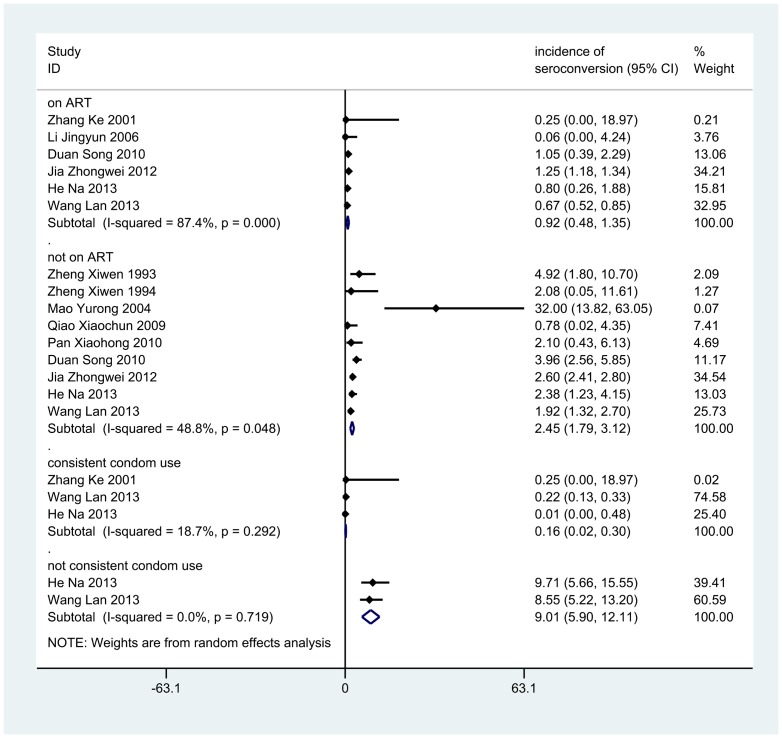
Forest plot of summary HIV transmission rates.

Among couples whose index partners were on ART, the pooled HIV seroconversion incidence rate was 0.92 per 100 PY (95%CI: 0.48, 1.35), and 2.45 per 100 PY (95%CI:1.79, 3.12) among couples with untreated index partners. The pooled HIV seroconversion incidence rate was 0.16 per 100 PY (95%CI:0.02, 0.30) for couples reporting consistent condom use, whereas couples reporting inconsistent use had an incidence of 9.01 per 100 PY(95%CI: 5.90, 12.11) (Figure 2).

### Prevention effectiveness of ART and consistent condom use

There were four studies in which the index-partner was stratified into ART use and non-ART use groups, and reported the seroconversion incidence of HIV-negative spouses of each group respectively, as these data were not adjusted by condom use level, so we pooled crude IRR between ART use group and non-ART use group, which was 0.47 (95%CI:0.43, 0.52).

**Figure 3:**
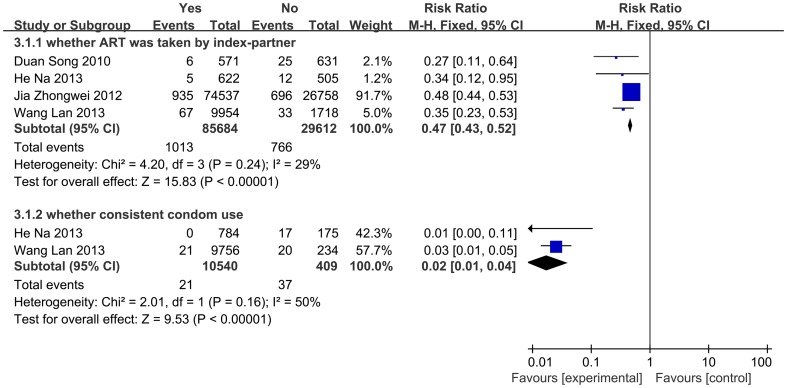
Incidence rate ratios for ART-stratified study estimates and condom use-stratified study estimates.

For the two studies which reported seroconversion incidence among consistent condom use group and not consistent condom use group, we found a pooled crude IRR of 0.02(0.01, 0.04) (Figure3).

### Prevention effectiveness of different combination of ART and condom use status

**Figure 4:**
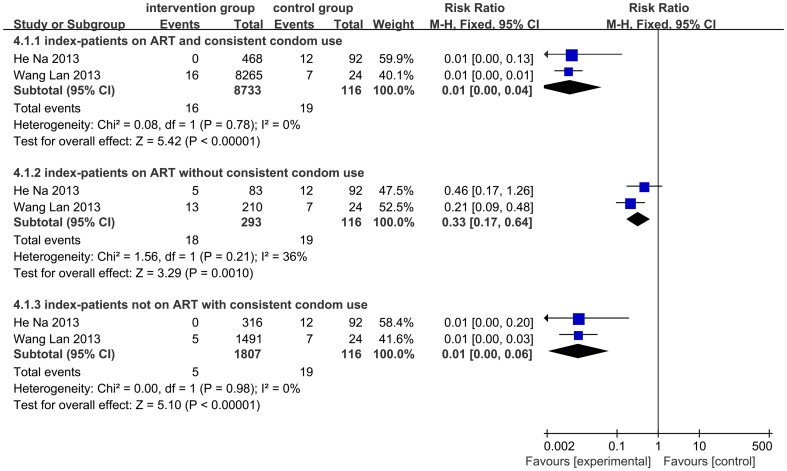
Incidence of rate ratios for three combinations of ART and condom use compare to no-ART and not consistent condom use couples.

There were two studies where we could extract seroconversion events and observation duration data under four combinations of ART and condom use levels, which is “index-partner on ART and consistent condom use” group, “index-partner on ART with not consistent condom use” group, “index-partner not on ART with consistent condom use” group and “index-partner not on ART with not consistent condom use” group. We made the “index-partner not on ART with not consistent condom use” group as the reference group, and found out that if the index-partner was on ART, the HIV-1 transmission risk was reduced by 67%, while the protective level could increase to 99% if the index-partner was on ART and the couple engaged in consistent condom use. (Figure 4).

### Effectiveness of ART stratified by follow-up time

We divided four studies which stratified couples by the index-partners' ART use status into two groups according to their follow up time based on the data analyzed, and for balance of each stratified group, we divided the studies based on whether couples were followed longer than 2 years. We found that the effectiveness of ART in preventing HIV transmission among the studies where follow up was equal to or more than 2 years was lower compared to the studies where the follow up time was less than 2 years. (IRR = 0.48 verse IRR = 0.29, Figure S1).

## Discussion

Our meta-analysis found out that the pooled HIV-1 seroconversion incidence rate estimates were lowest in the serodiscordant couples with consistent condom use, the crude IRR was 0.02(95%CI: 0.01,0.04) for consistent condom use versus not consistent condom use group, while a 53% reduction (95%CI: 48%, 57%) in HIV-1 sexual transmission with ART use by the index-partner from ART-stratified studies.

To assess the adjusted effectiveness for sexual HIV prevention of condom use, ART in index partners, or both methods among serodiscordant couples, we found that prevention effectiveness was highest among those using both prevention strategies. The results showed that ART reduced transmission in couples with imperfect condom use by as much as 67%, but in couples reporting “consistent condom use” simultaneous use of ART, the prevention level could increase to 99% (95%CI: 0.96,1.00). Therefore, ongoing condom use and safe sex education should remain a cornerstone of China's HIV control policy. As China promotes earlier and wider ART coverage as part of its new treatment as prevention policy for serodiscordant couples, it is important that advances in new efforts do not come at the cost of traditional condom promotion for HIV prevention.

It is worth noting that results from a Chinese national observational cohort study showed that the protection effectiveness of ART was only significant in the first year [28], and in our stratified analysis by follow up time of studies showed that the effectiveness of ART in preventing HIV transmission is lower in the longer follow up period group. That might be because the key to successful treatment as a prevention strategy is persistent stable viral suppression. However, unlike most of the settings where researchers showed protective effects of ART [11], [18], the Chinese HIV/AIDS patients were not managed under special conditions but were rather recipients of routine medical care. Most HIV/AIDS patients receiving ART at village clinics or township hospitals had higher rates of virologic failure compared to those patients treated at a county level hospital [29]. A cohort study in rural China showed that after 7 years of ART, 76.4% of patients experienced virologic failure and 64.4% patients had drug-resistant mutation [30]. These facts implied that durable and successful ART treatment outcomes are still serious challenges in China. Long term follow up studies are needed to figure out whether the “treatment as prevention” strategy would be feasible and durable in the long run.

Our study has several limitations. First, as in all longitudinal studies, our observations may be affected by left-censoring selection bias due to enrollment of “survivor” couples who remained discordant throughout the earlier and presumably risker phases of the discordant partnership. As a result our estimates of transmission incidence are likely biased towards the null. Second, there are other factors of HIV transmission besides ART use by the index-partners and condom use, such as gender of index-partners, HIV infection routes of the index-partners, sex activity frequency, and STD infection among couples which could not be factored into the current analysis.

## Supporting Information

Checklist S1 **PRISMA 2009 Checklist.**
(DOC)

Figure S1 **Effectiveness of ART in prevention HIV transmission stratified by follow up time.**
(TIF)
