# Supplementary figures and images for: Effectiveness of ART and Condom Use for Prevention of Sexual HIV Transmission in Serodiscordant Couples: A Systematic Review and Meta-Analysis
Source: PLoS One. 2014 Nov 4;9(11):e111175. doi: 10.1371/journal.pone.0111175 (PMC4219707; doi:10.1371/journal.pone.0111175)

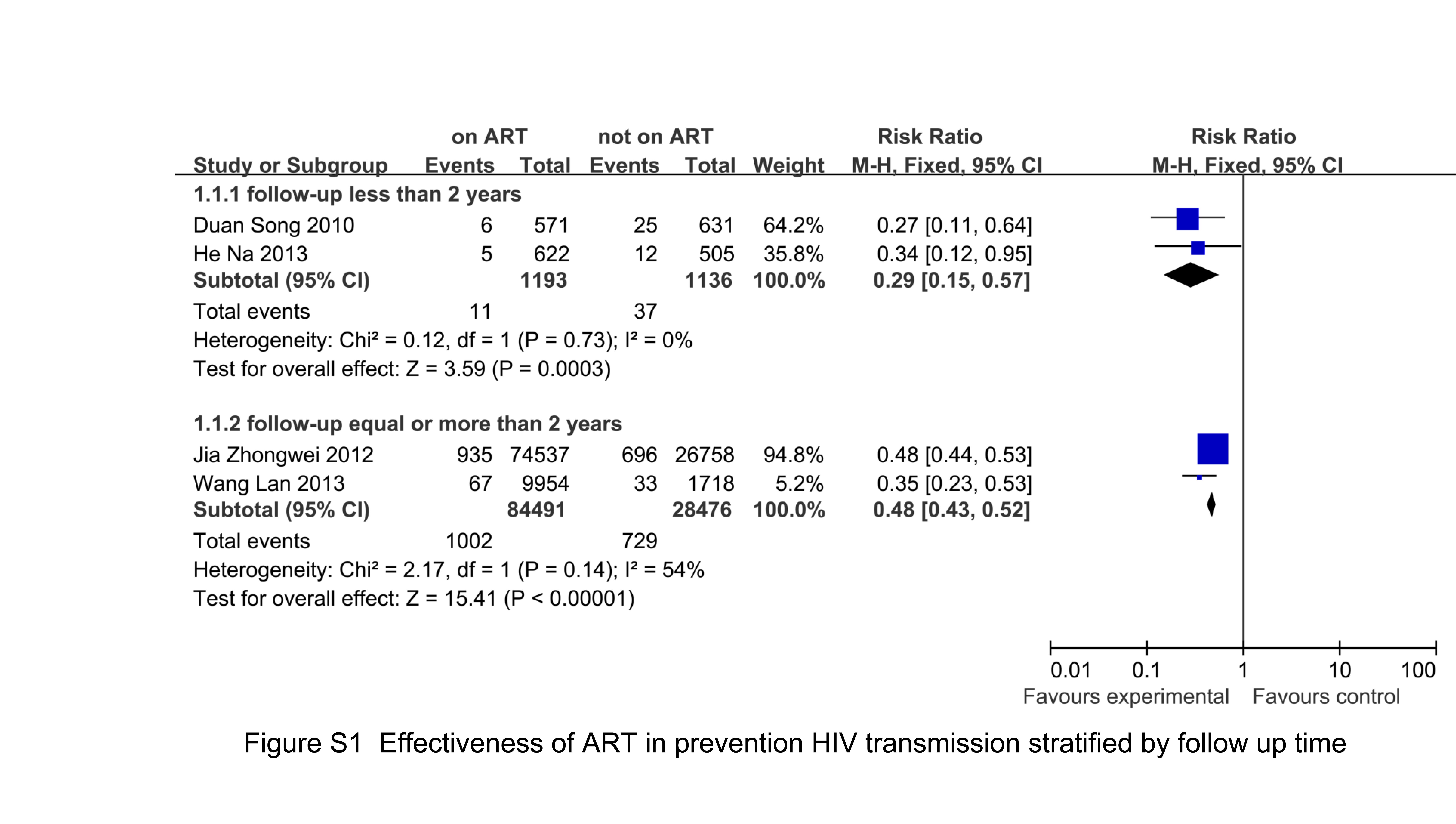

Supplement: Figure S1 — Effectiveness of ART in prevention HIV transmission stratified by follow up time. (TIF) [file pone.0111175.s002.tif]
